# Case report: Rhabdomyolysis in children in acute and chronic disease—a challenging condition in pediatric emergency medicine

**DOI:** 10.3389/fped.2023.1070465

**Published:** 2023-03-09

**Authors:** N. Mand, C. Donath, A. Leonhardt, S. Weber, M. Kömhoff

**Affiliations:** ^1^ Pediatric Intensive Care, Department of Pediatrics, Philipps-University Marburg, Marburg, Germany; ^2^ Pediatric Nephrology, Department of Pediatrics, Philipps-University Marburg, Marburg, Germany

**Keywords:** rhabdomyolysis, hereditary metabolic disease, LPIN1 mutation, circulatory failure, acute kidney injury (AKI)

## Abstract

Rhabdomyolysis is a challenging condition in pediatric emergency departments (PED): It ranges from asymptomatic illness with isolated elevation of creatine kinase (CK) levels to a life-threatening condition associated with extreme elevations in CK, electrolyte imbalances, circulatory failure (CF), acute kidney injury (AKI), and multi-organ disease. Most common causes of rhabdomyolysis are viral myositis and trauma, hereditary metabolic myopathies must be considered when facing rhabdomyolysis in early childhood. We report two cases of severe rhabdomyolysis with CF in our PED, thereby summarizing first-line management of rhabdomyolysis.

## Introduction

Rhabdomyolysis (RM) is characterized by (skeletal) muscle necrosis and subsequent release of its intracellular contents including serum creatine kinase (CK), myoglobin, potassium, and phosphorus into the blood (1). This occurs either due to direct muscle cell membrane damage or as a result of cellular energy depletion (2, 3). In the pediatric population, two thirds of all cases are caused by viral myositis and trauma. Other typical causes include metabolic disorders, exercise, and drug overdose (1, 3, 4).

More than 25,000 pediatric and adult cases are reported annually in the US (5). The exact incidence of pediatric RM is unknown, with many mild cases probably going unrecognized (2, 6).

In adults, RM is defined as a clinical syndrome of acute muscle weakness, myalgia, and muscle swelling combined with a CK of >1,000 IU/L or higher than five times the upper limit of normal in the absence of significant elevations of brain or cardiac CK fractions (1, 7). There is a wide variation in the clinical presentation of RM (8). The level of CK, however, does not predict the severity of symptoms (4).

The most common symptoms in childhood are myalgia, weakness, and fever, up to one-third present with convulsions and/or reduced consciousness, while dark urine is rather rare (1, 4, 9). Infants and young children may present with nonspecific symptoms such as vomiting or apathy (9), thus making RM a challenging diagnosis in pediatric emergency departments (PED). Severe complications include electrolyte imbalances, circulatory failure (CF), disseminated intravascular coagulation, and hepatic dysfunction (8). Up to 10% of the patients develop acute kidney injury (AKI) (1, 4). A weak correlation between peak CK values and the incidence of AKI has been reported (10). In the absence of other significant risk factors such as sepsis the risk to develop AKI starts to increase above CK levels of 15.000 IU/L (10).

We report two cases of severe rhabdomyolysis with CF in our PED developing AKI necessitating renal replacement therapy further on.

## Case presentations

### Case 1

**Figure 1:**
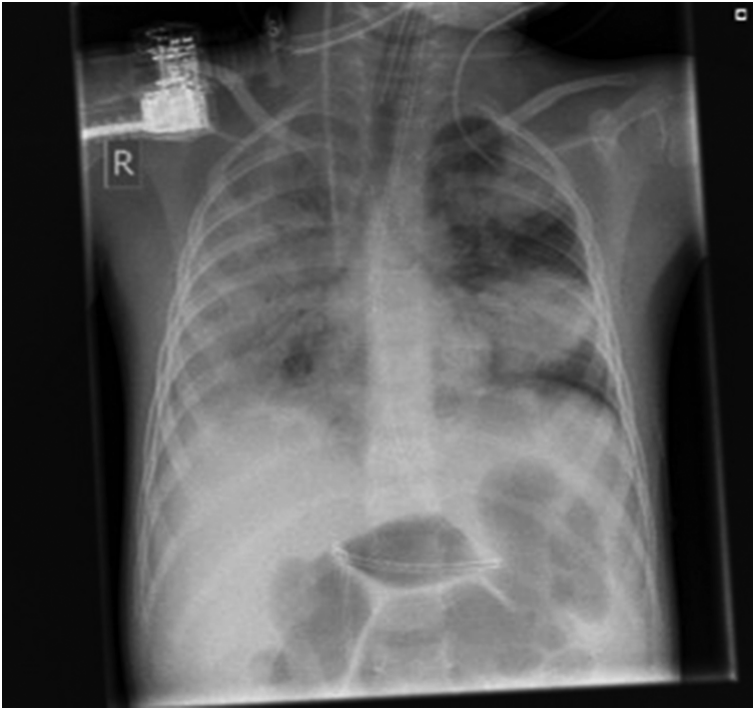
X ray case 1, twelve hours after admission.

A previously healthy 4-year-old boy was introduced with malaise, fever, airway infection until 3 days ago, and myalgia. His mother reported increasing weakness, unwillingness to walk, decreasing diuresis and acute somnolence on the day of presentation. In the PED he showed signs of decompensated hypovolemic shock [CRT 4 s, HR 100 bpm, BP 72/28 (58) mmHg, GCS 10, Temp. 34.4°C]. Thus, 20 ml/kg crystalloids were infused manually within five minutes, and another 40 ml/kg crystalloids within the first hour on the PICU. The boy improved transiently. Capillary POCT analysis showed metabolic acidosis and hyperkalemia (pH 7.12, potassium 10.2 mmol/L, lactate 7.4 mmol/L, Hb 16.4 g/dl). Approximately 3 h after admission the patient went into ventricular tachycardia (VT) which was terminated with calcium gluconate within 2 min. Laboratory results confirmed hyperkalemia of 8 mmol/L and a CK > 100,000 U/L. Beyond that, inflammation markers were slightly, while liver enzymes were markedly elevated (AST 4,328 U/L), clotting was normal. Despite extensive conservative measures to treat hyperkalemia (bicarbonate, albuterol, and insulin-glucose-infusion) three more episodes of VT occurred. Therefore, continuous veno-venous hemofiltration (CVVH: Filter: FX50, Fresenius Medical Care, Bad Homburg, Germany; initial settings: bloodflow 60 ml/min, dialysis 2,500 ml/h) was initiated (serum creatinine at this point 0.65 mg/dl, urea 77 mg/dl, phosphate 3.2 mmol/L). In addition, catecholamine therapy with epinephrine, dopamine, and norepinephrine was begun due to persisting cardio-circulatory failure despite ongoing volume substitution with normal saline, human albumin, and fresh frozen plasma. The patient was intubated and pressure-controlled ventilated with NO due to developing clinical and radiological signs of ARDS (see Figure 1) (min. PaO_2_/FiO_2_ 60; OI max. 38, max. PEEP 16 cm H_2_O, max. driving pressure 21 cm H_2_O). Sedation was initiated with midazolam and ketamine and sustained with midazolam and fentanyl. Broadband antibiotics were started.

**Figure 2:**
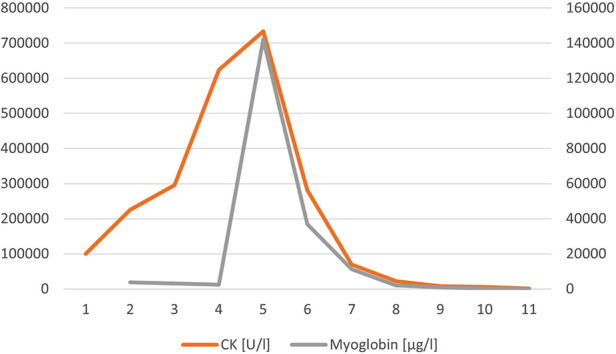
CK and myoglobin in case 1.

During the following days, the boy's respiration and circulation stabilized while diuresis ceased on day 2. CK and myoglobin continued to increase till day 5 (max. CK 734,098 U/L, max. myoglobin 142,121 U/L, see Figure 2), and electrolytes normalized during CVVHDF. There were no further episodes of VT. Starting from day 4, catecholamines were slowly tapered, and respiratory weaning was begun. Initial hepatic dysfunction (max. AST 13,913 U/L on day 5) and intravascular coagulation normalized gradually. The boy was extubated on day 8, CVVHDF was discontinued on day 12 (max. serum creatinine 3.3 mg/dl), and he was then transferred to the pediatric ward. Intermittent HD was terminated on day 20 and retention parameters remained normal. With tailored exercise multiple times daily muscle weakness in arms and legs slowly decreased. The boy was discharged from the hospital on day 27, CK still being mildly elevated (791 U/L).

Influenza A was identified as the cause of this severe rhabdomyolysis *via* PCR-testing. Further extensive testing for other viruses, bacteria, drug toxins, and metabolic myopathies remained negative.

So far, the boy did not redevelop any other RM episode. Being 8 years now, renal function is normal, he has no cognitive or motoric impairments, and he is attending a regular school and is playing soccer several times a week.

### Case 2

**Figure 3:**
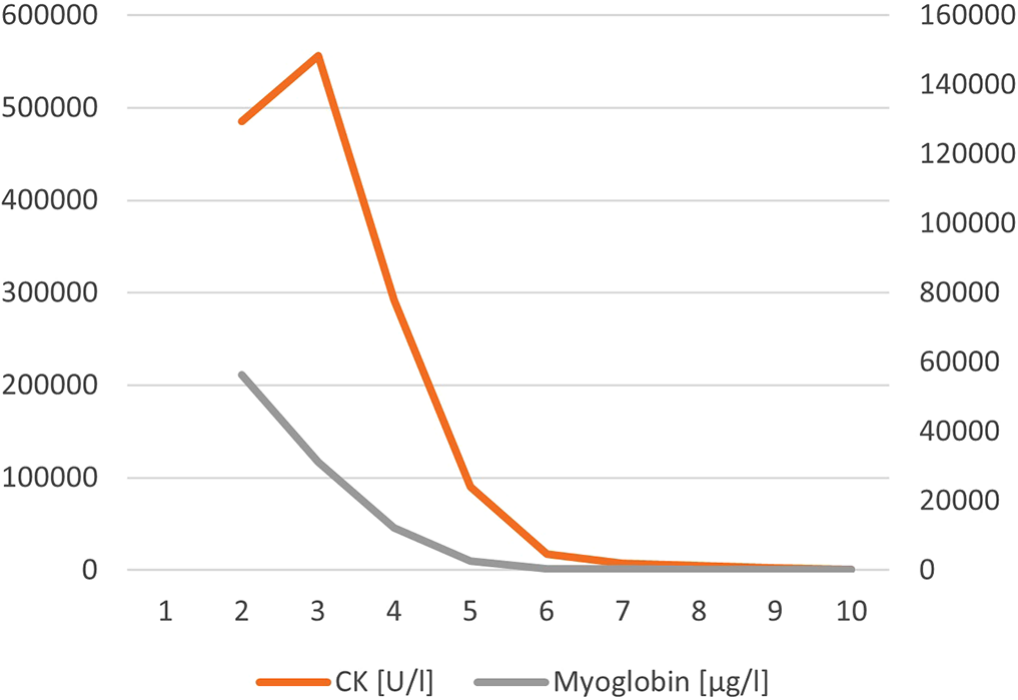
CK and myoglobin in case 2

An 11-month-old male infant with a known motor development delay but otherwise healthy was admitted to the PED with recurrent vomiting over the last two days. No fever was reported, but mild diarrhea, and increasingly dry diapers. Physical examination revealed an ubiquitous sensitivity to touch and signs of a hypovolemic shock [HR 156 bpm, BP 112/66 (87) mmHg, RR 64 pm, Temp. 36.5°C, GCS 11]. Arterial POCT analysis revealed normokalemia with respiratory compensated metabolic acidosis (pH 7.4, pCO_2_ 28 mmHg, HCO_3_ 17.5 mmol/L, BE −5.5 mmol/L, potassium 4.9 mmol/L). Laboratory results showed markedly elevated CK and liver enzymes (CK 343,030 U/L, AST 9,365 U/L, see Figure 3). Despite volume substitution with 50 ml/kg crystalloids and normal saline anuria persisted and continuous veno-venous hemofiltration (CVVH: Filter: FX40, Fresenius Medical Care; initial settings: bloodflow 60 ml/min, dialysis 2,500 ml/h) was started on day 2 when CK was peaking (max. 556,386 U/L). Additionally, alkalinization of urine was achieved with bicarbonate. Serum creatinine, urea, and electrolytes stayed normal throughout the hospital stay, while CK and myoglobin decreased gradually. CVVHF was discontinued on day 12 when adequate diuresis was re-established. During recovery, CK-levels dropped markedly, but did not normalize completely. The patient was discharged with slightly elevated CK (1,673 U/L) on day 17; subsequently, CK levels peaked maximally 1,500 U/L during febrile disease. A metabolic myopathy was suspected and genetic alterations consistent with compound heterozygeous, pathogenic mutations in *LPIN1*, causing acute, recurrent rhabdomyolysis (OMIM #268200) were detected in the patient and his parents: Sequencing of DNA from the patient and his parents revealed a pathogenic splice site mutation (c.2513 + 1G > A) in the patient and his mother but none in the father. Due to the high degree of suspicion of a second mutation in *LPIN1* in the patient and hence possibly also in his father, mRNA sequencing from RNA isolated from paternal fibroblast were performed. This approach revealed that exon 3 of *LPIN1* was reduced by approximately 50%, resulting from a cryptic splice site mutation in *LPIN1*. Importantly, in the second child of the family who carries the maternal mutation, mRNA sequencing excluded a splicing defect. A third child was conceived without mutation in *LPIN1* following preimplantation genetic diagnosis.

Children bearing bi-allelic mutations in *LPIN1* which encodes an enzyme important in the pathway of triglyceride and phospholipid biosynthesis, are prone to recurrent bouts of rhabdomyolysis especially in states of catabolic stress but sometimes also without overt precipitating factors (11). The parents were instructed to administer an oral carbohydrate-electrolyte-rehydration-solution whenever fever or fasting occurred. At 2.5 years of age, while on holiday, another episode with rapid deterioration occurred in our patient resulting in cardiac arrest. Due to massive spasms of the masseter, airway securement was not possible. The boy died after 60 min of unsuccessful cardiopulmonary resuscitation.

## Discussion

Rhabdomyolysis in children has a broad spectrum of causes and severity and is potentially life-threatening. The heterogeneity of its symptoms makes it a challenging condition to diagnose and treat in the PED (2, 4).

Besides trauma, infections are the main cause of rhabdomyolysis in children, with mycoplasma spp. and different viruses such as influenza, enteroviruses, and SARS-CoV-2 being known triggers (1, 4, 12, 13). In our first case, influenza A was identified, which is the most common viral trigger for RM (1). Influenza-associated myositis is mostly a benign complication and typically occurs, when symptoms of influenza are about to resolve (14). A progression into RM should be suspected if muscle pain is worsening or severe enough to discourage walking (see Table 1) (14, 15). The classic triad of RM-symptoms including muscle pain, weakness, and dark urine is rarely seen in children (1, 4, 9). Especially infants and young children present atypically with fever, vomiting, and convulsions, thus being at risk of being misdiagnosed as simple gastroenteritis or, if symptoms are more severe, an intracranial infection (9). In our second case gastroenteritis causing hypovolemic shock was suspected initially. Routine laboratory results revealed markedly elevated CK and liver enzymes, thus prompting investigations for a hereditary metabolic myopathy. Any episode of severe RM, especially in infants or recurrent episodes of RM triggered by minimal exercise at any age should be a reason to suspect hereditary diseases (9, 16, 17). Genetic causes of RM include metabolic muscle disorders (e.g., fatty acid metabolism disorders or abnormal glycogen storage), mitochondrial disorders, disorders of intramuscular calcium release, and muscular dystrophies (16, 18). With autosomal-recessive mutations in the *LPIN1* gene causing intracellular energy deficiency with severe RM episodes poor prognosis and high mortality have been reported (16–18). As a frequent cause of early-onset RM *LPIN1*-mutation was detected in our second case subsequently.

**Table 1:** Clinical suspicion for rhabdomyolysis (“red flags”) that should prompt evaluation of CK.

- Myalgia and/or muscle weakness (“unwillingness to walk”) - Tea-colored urine, decreased urine output (“dry diapers”) - Signs of hypovolemic shock - Fever, vomiting and lethargy in infants - Above mentioned symptoms after low-intensity exercise or fasting - Hyperkaliemia and/or hypocalcemia

In both patients lethargy and severe hypovolemia were the leading symptoms prompting initial therapy. Significantly elevated CK, myoglobin and liver enzymes were detected in routine laboratory results. In one patient POCT analysis revealed typical electrolyte disbalances thus determining the subsequent therapeutic approach. The pathological hallmark of RM is necrosis of muscle cells due to an insult to the cell's membrane or cellular energy depletion (2). This is contrary to myositis, where the muscle is inflamed but cell walls remain intact. Compromised cell integrity leads to the leakage of cellular contents into the circulatory system and energy deficiency on electrolyte transporters (e.g., Na^+^/Ca^2+^ or Na^+^/K^+^ exchanger) leads to massive shifting of electrolytes (2, 8). Initially, serum levels of potassium and phosphate increase as these components are released from the cells, and serum concentrations of calcium are decreased as calcium moves into the cells aggravating the destruction of cell membranes (2, 8). Intracellular proteins (CK, myoglobin, lactate dehydrogenase (LDH), aminotransferase (AST), aldolase) are released into the bloodstream (6, 8). CK typically rises within 12 h of the onset of muscle injury, peaks within 1–3 days, and declines 3–5 days after the cessation of muscle injury (8). Unlike haemoglobin, which is avidly bound by haptoglobin, myoglobin levels rapidly exceed the unspecific protein-binding capacity of plasma, resulting in a dark red-brown colored urine and precipitation in the glomerular filter, thus facilitating AKI (6, 10).

AKI is a major mortality factor in children with or without RM (10, 19). Children with AKI as a component of multisystem failure have a much higher mortality rate than children with intrinsic renal disease (20, 21). In critically ill children severe AKI occurs in up to 15% (21, 22). Reported incidence in children with RM highly varies, rates between 5% and 42% have been described (1, 10, 23). Sequestration of water in injured muscles initiates volume depletion (10, 24). Clinical findings like dry mucous membrane, decreased skin turgor, sunken anterior fontanelle, tachycardia, or hemoconcentration should prompt fluid therapy, as AKI is more likely to develop in the presence of dehydration (10, 25). In addition, precipitating myoglobin and myoglobin-mediated tubular cytotoxicity, renal vasoconstriction/hypoperfusion as a result of circulatory impairment and consequent metabolic acidosis is probably contributing to AKI (25). Positive urinary heme dipstick results are an earlier indicator of potential AKI than creatinine and urea might be (1, 10), CK-levels being an indicator for AKI only in adult traumatic RM (4, 26). Attempts to remove myoglobin *via* extracorporeal therapy before AKI has established were successful in case reports (27, 28), but its benefits could not be clearly confirmed in large trials (10). This notion is supported by a recently published trial describing the effects of high cutoff vs. conventional renal replacement therapy in 70 adults with AKI and rhabdomyolysis. Even though myoglobin clearance with continuous veno-venous hemodialysis using high cutoff dialyzer was significantly higher compared to control, there was no clinical benefit and ICU-mortality was even significantly higher in the “high cutoff” group (29).

Hence, irrespective of the underlying aetiology, the main therapeutic intervention in rhabdomyolysis is the aggressive administration of intravenous fluid to avoid circulatory failure (CF) and AKI (see Table 2) (4). In children with recognized shock volume resuscitation with one or more early fluid boluses of 10 ml/kg crystalloids have to be performed, up to 60 ml/kg might be needed in the first hour of treatment (30). Reassessments after each bolus are aimed at early recognition of circulatory improvement or signs of fluid overload and cardiac failure (30). Severely volume-depleted children might need up to 100 ml/kg fluids within the first 8 h of presentation (30). As long as kidney function is intact, volume therapy after resolution of hypovolemic shock is aimed at a high urine output to dilute and eliminate the heme protein; diuretics are applied in states of volume overload (24, 28).

**Table 2:** Rhabdomyolysis treatment in the PED.

1. Evaluation of the critical ill child (ABCDE approach), proper management of airway, oxygenation and ventilation. 2. Fluid resuscitation when signs of hypovolemic shock (CRT↑, HR↑/BP↓, lactate↑, low urine output, GCS↓, temp. ↓): one or more crystalloid bolus(es) of 10 ml/kg, up to 40–60 ml/kg in the first hour. 3. Fluid therapy when no signs of hypovolemic shock: 10% dextrose in normal saline at 1.5–2 times maintenance. 4. Early POCT-analysis: watch for high potassium, low calcium. Correct electrolyte abnormalities. In acute life-threatening hyperkalemia give calcium iv (e.g., calcium gluconate 10% 0,5 ml/kg max. 20 ml). 5. Consider ECG: watch for high *T* waves. 6. Urinary dipstick: consult pediatric nephrology when heme positive. 7. Avoid nephrotoxic medication, consider dialysis.

There is conflicting evidence on use of mannitol and sodium bicarbonates to alkalinize the urine (24). Myoglobin excretion is enhanced at a urine pH of 8.0 (31). However, studies in children are lacking (1, 24). Sodium bicarbonate (1 mmol/kg IV, repeat as necessary) should be used in the presence of hyperkalemia and metabolic acidosis (pH < 7.2), though, the effect is slow (30). In life-threatening hyperkalemia intravenous calcium (e.g., calcium gluconate 10% 0.5 ml/kg, max. 20 ml) is administered, the effect occurring within minutes and lasting for up to an hour. Insulin-glucose-infusion and nebulized beta-agonists are additional conservative measures to decrease potassium levels (30). Hyperkalemia as the first “red flag” of increased cell turnover can be easily detected in POCT analyses but may not be present initially. ECG abnormalities such as high-amplitude *T* waves can precede hyperkalemia, especially in hereditary myoglobinuria and should prompt aggressive treatment without delay. Progression to critical hyperkalemia initiates cardiac dysrhythmias and possibly cardiac arrest (2, 8, 17). Other potentially lethal electrolyte disturbance such as hypocalcemia or severe metabolic acidosis should be corrected as early as possible (4, 24).

In hereditary metabolic disorders, such as in autosomal recessive recurrent myoglobinuria caused by gene mutations in *LPIN1* (OMIM *605518) presented by case 2, the intramuscular energy deficiency that is associated with RM is effectively treated by hyperhydration and energy supply, using high-concentration glucose-solutions next to crystalloids (32). Consequently, parents should be instructed to maintain a high caloric intake in situations with increased energy demand (32, 33). Concomitantly, dexamethasone can be successfully added in the standard treatment (33).

## Conclusion

Rhabdomyolysis can present as a life-threatening condition in the pediatric emergency department. Patients with a history of myalgia and/or muscle weakness, a decreased urinary output or with signs of hypovolemic shock should prompt an evaluation for CK. Hyperkalemia as the first “red flag” of RM can be easily detected in POCT analyses, which should be performed early in every critical patient. Urinary dipstick is an easily available and non-invasive screening test for the identification of patients who need monitoring of renal function (1, 34). In children, rhabdomyolysis is most commonly caused by viral disease or trauma (1). Nevertheless, genetic disorders such as hereditary metabolic myopathies have to be excluded especially if the first episode occurs in early childhood or in subjects with extremely elevated CK values (18). Until the establishment of a genetic diagnosis, which is critical because of the high risk of recurrence, and upon confirmation of a genetic diagnosis, respectively, sufficient administration of carbohydrates and fluids in myoglobulin precipitating conditions (febrile disease, fasting, strenuous exercise) is potentially lifesaving.

## Data Availability

The original contributions presented in the study are included in the article/Supplementary Material, further inquiries can be directed to the corresponding author.
